# Opioid Use and Prescription Opioid Use Disorder: Biopsychosocial Characterisation of a Clinical Chronic Pain Cohort

**DOI:** 10.1002/ejp.70081

**Published:** 2025-07-19

**Authors:** Sofia Wagner, Hanna Ljungvall, Hedvig Zetterberg, Rolf Karlsten, Lisa Ekselius, Pernilla Åsenlöf

**Affiliations:** ^1^ Department of Women's and Children's Health Uppsala University Uppsala Sweden; ^2^ Uppsala University Hospital Uppsala Sweden; ^3^ Department of Surgical Sciences Uppsala University Uppsala Sweden; ^4^ Department of Women's and Children's Health Upsala University, WOMHER Uppsala Sweden

## Abstract

**Background:**

This study aimed to examine: (a) characteristics associated with long‐term opioid use and (b) characteristics associated with problematic opioid use, here defined as prescription opioid use disorder (P‐OUD), in patients referred to specialised pain care.

**Methods:**

This cross‐sectional study utilised baseline data from a clinical chronic pain cohort. Eligible participants included adults > 18 years old, not undergoing cancer treatment, had pain > 3 months, and had been referred to a specialised pain care centre in Sweden. Bivariate logistic regression and multivariable logistic regression with forward selection were used to examine the associations between biopsychosocial variables and either long‐term opioid use or P‐OUD.

**Results:**

Of the 339 patients included, 194 (57%) were using opioids, 159 (47%) had long‐term opioid use (> 90 days), and 34 (21% of those with long‐term opioid use) had P‐OUD. Longer pain duration, unemployment, more pain catastrophising, lower health‐related quality of life, and worse balance increased the likelihood of long‐term opioid use. Long‐term use of high doses was associated with a greater prevalence of psychiatric and cognitive‐behavioural problems compared to long‐term use at low to moderate doses. Long‐term opioid use, younger age, trauma exposure, more pain catastrophising, and fear of movement increased the likelihood of P‐OUD.

**Conclusions:**

Our results demonstrate the importance of identifying and treating salient factors that may sustain both the pain condition and long‐term opioid use. Many of the biopsychosocial variables associated with long‐term opioid use and P‐OUD can be addressed within interdisciplinary pain management and rehabilitation programmes.

**Significance:**

This study is part of the U‐PAIN cohort study on a clinical sample from a highly specialised pain centre in Sweden. It is based on a deep and thorough characterisation of patients referred to the clinic to evaluate risks and benefits of opioid therapy in chronic pain. It adds valuable information on the complexity of opioid therapy in chronic pain; the results highlight the need for interdisciplinary multimodal evaluation and treatment.

## 1 Introduction

Opioids have been used since ancient times to alleviate pain and induce euphoria; however, they are also among the drugs most commonly associated with addiction and unintentional drug overdoses worldwide (Martins et al. 2015). Opioids are not recommended as a first‐line treatment for any chronic pain condition, that is, non‐malignant pain lasting > 3 months, and are not at all recommended for nociplastic pain conditions (Dowell et al. 2022; Kosek 2024). Instead, multimodal treatment strategies targeting chronic pain as a biopsychosocial condition in which combinations of biological, psychological, and social factors contribute to the chronicity of pain should be provided (Borsook et al. 2018; Cohen et al. 2021; Gatchel et al. 2007). Opioids should only be considered for treating chronic non‐malignant pain when other treatments are intolerable or ineffective and after a thorough risk–benefit assessment has been conducted (Häuser et al. 2021). This is because opioids, especially long‐term opioid therapy (LTOT) > 3 months, are associated with several dose‐dependent risks. LTOT is associated with impaired cognition, increased morbidity and mortality, overdose‐related deaths, opioid dependence, that is, the development of tolerance and withdrawal, and prescription opioid use disorder (P‐OUD) (Ballantyne 2012; Nury et al. 2022). OUD is a psychiatric diagnosis defined as opioid use leading to impairment or distress as manifested by at least two out of 11 symptom‐based criteria within a 12‐month period. These symptoms include loss of control regarding opioid intake and craving for the opioid. P‐OUD refers to the used opioid being prescribed (American Psychiatric Association 2013).

Chronic pain, especially together with LTOT, is associated with psychiatric co‐morbidity, and both pain and psychiatric problems are risk factors for P‐OUD, adding risk for patients with comorbidity. Other known risk factors for P‐OUD include past or current substance use disorders (SUD), heritability, younger age, mental health problems, and receiving opioids from several prescribers (Campbell et al. 2020; Cragg et al. 2019). However, detecting P‐OUD in patients with chronic pain is challenging, given that the opioid use often is driven by the search for pain relief and coping with internalising conditions (e.g., affective symptoms), rather than drug craving and loss of control (Manhapra et al. 2020).

There is a lack of high‐quality studies applying the biopsychosocial model examining which variables are associated with LTOT and P‐OUD among patients with chronic pain (Voon et al. 2017). Therefore, the Uppsala pain cohort (U‐PAIN), with the overall aim to identify predictors for risks and benefits associated with long‐term opioid use, was established. The cohort will provide a cross‐sectional study of baseline characteristics of non‐opioid users and opioid users, and a 5‐year longitudinal study with endpoint measurements every 12 months to prospectively identify predictors and trajectories of opioid use. Variables relevant to chronic pain aligned with the biopsychosocial model (Cohen et al. 2021; Gatchel et al. 2007) will be mapped because of their hypothesised association with opioid use and P‐OUD in patients with chronic pain treated in specialised pain care (Ljungvall, Lind, et al. 2022).

This is a cross‐sectional study aimed at examining: (a) characteristics associated with long‐term opioid use and (b) characteristics associated with problematic opioid use, defined as P‐OUD, in patients receiving treatment in specialised pain care.

## 2 Methods

As part of the U‐PAIN cohort, the study was approved by the Swedish Ethical Review Authority's regional ethics board in Uppsala (EPN Uppsala D‐No 2016‐376, 2020‐05283). Written informed consent was provided before study inclusion and data collection, in accordance with the Helsinki Declaration of 1975. This study has a cross‐sectional design, including data collected at baseline and complies with STROBE reporting guidelines (von Elm et al. 2014). A detailed description of the methodology, sampling strategies, and procedures is provided in Ljungvall, Lind, et al. (2022) and ClinicalTrials.gov (ID: NCT06063902; 03/10/2023).

Eligible participants were adults ≥ 18 years, competent in Swedish, not undergoing cancer treatment, and seeking treatment for non‐malignant chronic pain > 3 months.

Participants were recruited between June 2018 and December 2022 from a Swedish university hospital's pain centre, which offers specialised multidisciplinary care nationwide. Trained staff screened and informed eligible individuals, who were invited to a research visit during their clinical consultation or inpatient stay. Participants completed online questionnaires, structured interviews, physical tests, and additional study‐specific questionnaires. Those not using the digital platform received paper versions.

### 2.1 Outcomes and Measures

Outcome measures were opioid use and P‐OUD. All opioids were converted into oral morphine milligram equivalents (MME) (Hallberg 2018). Opioid use was categorised as: no use, short‐term, or intermittent use (opioids use for < 3 months or intermittent use as dosing up to 10 days/months, independent of time frame), long‐term use at low to moderate doses (opioids ≥ 3 months, more than 10 days/month and mean daily dose < 100 mg MME), long‐term use at high doses (opioids for ≥ 3 months, ≥ 100 MME). Criteria for the categories were based on current evidence of dose‐dependent risks and dosing recommendations in clinical guidelines (Dowell et al. 2022; Manchikanti et al. 2017; Swedish Medical Products Agency 2017). Opioid use was measured using the Time Line Follow Back interview (TLFB). The TLFB is used for retrospective self‐reported information about substance use behaviour and has been found feasible and reliable in this specific population (Carey et al. 2004; Ljungvall, Lind, et al. 2022).

P‐OUD was assessed according to the DSM‐5 criteria (American Psychiatric Association 2013), using a modified version of the substance use disorder (SUD) section (J) of the Swedish Mini‐International Neuropsychiatric Interview (M.I.N.I.) for DSM‐5. Here, the behavioural criteria were not considered positive if they only were related to pain impairment and the opioids were used as prescribed (Ljungvall, Lind, et al. 2022). P‐OUD can be mild (2–3 symptoms), moderate (4–5 symptoms), or severe (≥ 6 symptoms). Symptoms of tolerance or withdrawal did not apply if the opioid was taken under medical supervision, that is, taken as prescribed. Moderate and severe P‐OUD equal opioid dependence, according to ICD‐10 (Degenhardt et al. 2015).

Variables for patient characterisation were chosen to capture inherent biopsychosocial constructs, such as pain, pain interference, and self‐rated health. Other targets are more specific domains: biological (e.g., age, sex, physical capacity), psychological (e.g., psychiatric symptoms, cognitive‐behavioural variables), and social (sociodemographic variables and social support). The multidimensional approach enables a comprehensive understanding of patient outcomes within the biopsychosocial model and in line with the Initiative on Methods, Measurement, and Pain Assessment in Clinical Trials' (IMMPACT) recommendations for chronic pain trials (Dworkin et al. 2008).

Individual and demographic variables were measured with a study‐specific self‐report form. Employment was self‐reported and included as having a job or studying. Social support was measured using the Swedish version of the Medical Outcome Study Social Support Survey (MOS‐SSS), which contains 19 items and constitutes four subscales: emotional or informational support, tangible support, affectionate support, and positive social interaction. Scale scores are then transformed to a 0–100 scale, with higher values indicating more support (Sherbourne and Stewart 1991). The MOS‐SSS has been found to be a valid and reliable tool in this particular population (Ljungvall et al. 2023).

The respondent's self‐rated health status was measured using the EQ‐5D‐5L visual analog scale (EQ VAS), where health is rated on a scale from 0, worst imaginable health, to 100, best imaginable health. The EQ VAS has demonstrated reliability and significant correlation with health‐related quality of life in a primary care population and adequate construct validity across diverse populations (Cheng et al. 2021; Xu et al. 2024). Smoking and other nicotine use were measured using the Swedish National Board of Health and Welfare indicator questions (National Board of Health and Social Welfare, n.d.). Hazardous alcohol use was measured using the Alcohol Use Disorders Identification Test (AUDIT) (Reinert and Allen 2007). Cut‐off values of 8 and 6 points for men and women, respectively, indicate hazardous alcohol use (Bergman and Källmén 2002). Illicit drug use was assessed using the M.I.N.I., section J.

Pain severity and pain interference were measured using the Brief Pain Inventory Short‐Form (BPI‐SF), where pain is reported at its *worst*, *least*, *on average*, and *now*; moreover, the response scale ranges from 0, no pain, to 10, pain as bad as you can imagine. The interference subscale of the BPI‐SF assesses how pain impacts seven constructs: mood, enjoyment of life, relationships with others, sleep, general activity, walking ability, and work, ranging from 0, does not interfere, to 10, completely interferes. A composite score for pain severity and pain interference was computed by the mean of the items on each scale. The level of pain relief due to treatment or medication is reported on a scale ranging from 0, no relief, to 100, complete relief (Cleeland and Ryan 1994). The BPI‐SF has been confirmed as a useful generic multidimensional pain outcome measure regarding internal consistency, criterion‐convergent validity, structural validity, and responsiveness in pain‐related musculoskeletal conditions (Jumbo et al. 2021). Pain duration was self‐reported. ICD‐11 chronic pain diagnoses were assessed retrospectively based on data from the medical records for the chronic pain classification.

For psychiatric symptoms, several different self‐report forms were used. Symptoms of depression were assessed using the Swedish version of the Patient Health Questionnaire 9‐item depression module (PHQ‐9), with a total score of 1–27 (1–4, minimal; 5–9, mild; 10–14, moderate and 20–27, severe levels of depression). The Swedish PHQ‐9 is found to be reliable, with high internal consistency and a stable factor structure in different patient populations (SBU—Swedish Agency for Helth Technology Assessment and Assessment of Social Services 2018). Symptoms of anxiety were measured with the Generalised Anxiety Disorder 7‐item scale (GAD‐7), with a total score of 0–21 (0–4 = minimal, 5–9 = mild, 10–14 = moderate, and 15–21 = severe levels of anxiety). The GAD‐7 is a valid and reliable screening tool for assessing anxiety disorders in primary care populations (Spitzer et al. 2006).

For the assessment of symptoms of attention deficit hyperactivity disorder (ADHD), the ADHD Self‐Rating Scale (ASRS‐v1.1) was used. The ASRS‐v1.1 screens adults for ADHD according to the DSM‐IV criteria (American Psychiatric Association 1994). The scale consists of 18 questions that are rated on a scale from 0, never, to 4, very often, and then dichotomised. A summed score > 9 is considered a positive screen for ADHD (Kessler et al. 2005).

Symptoms of insomnia were measured using the Insomnia Severity Index (ISI), with a total score of 28 (0–7 = no clinically significant insomnia, 8–14 = sub‐threshold insomnia, 15–21 = moderate insomnia, and 22–28 = severe insomnia). The ISI is a valid and reliable measure of perceived insomnia (Bastien et al. 2001).

Exposure to traumatic life events was measured using the Life Event Checklist (LEC‐5), a widely used self‐report measurement of prior trauma exposure that was designed to accompany the Clinician Administered PTSD Scale (CAPS). The LEC consists of 17 questions about different traumatic events, including exposure to physical and sexual abuse, asked in reference to the proximity to exposure (experienced, witnessed, or learned about). We only summarised the experienced traumatic events, with a total score ranging from 0 to 17. This scoring method has shown good reliability in traumatically injured civilian samples (Weis et al. 2022).

Personality was assessed using the Swedish University Scales of Personality (SSP) consisting of 91 items. Each item is a statement that is rated on a four‐point scale, from does not apply at all to applies completely. The items are subdivided into 13 subscales, and summated scale means are calculated for the three factors of neuroticism, aggressiveness, and extraversion. The SSP has satisfactory internal consistency and face validity in a general population (Gustavsson et al. 2000).

SUDs other than alcohol were assessed using the M.I.N.I, section J. For SUD, two to three symptoms indicate mild SUD, 4–5 symptoms indicate moderate SUD, and ≥ 6 symptoms indicate severe SUD syndrome (American Psychiatric Association 2013).

For cognitive‐behavioural variables, pain catastrophising was assessed using the Pain Catastrophising Scale, Swedish version (PCS‐SW). Each item scores from 0, never, to 4, all the time, with a total score ranging from 0 to 52. PCS‐SW has demonstrated good reliability and validity in individuals with chronic pain (Kemani et al. 2019).

Fear of movement or (re)injury was measured with the Swedish version of the Tampa Scale of Kinesiophobia 11‐item scale (TSK‐11). Each item ranges from 1, strongly disagree, to 4, strongly agree, with a total score ranging from 1 to 44. The TSK demonstrates acceptable reliability and validity in chronic musculoskeletal pain patients (Larsson et al. 2014; Roelofs et al. 2007).

Pain self‐efficacy was assessed using the Pain Self‐Efficacy Self‐Rating 2‐item scale (PSEQ‐2). The scale rates the respondents' confidence in performing activities despite the presence of pain on a 7‐point scale from 0, not at all confident, to 6, completely confident, with a total score ranging from 0 to 14. PSEQ‐2 has demonstrated acceptable reliability and validity in chronic pain patients (Nicholas et al. 2015).

Injustice was measured using the Injustice Experience Questionnaire (IEQ‐S). IEQ‐S addresses perceived injustice following an injury and consists of 12 items. Respondents indicate their agreement on a five‐point scale from 0, not at all, to 4, all the time. The total score is calculated by adding the scores of each item, and a total score of ≥ 30 indicates clinical relevance (Sullivan et al. 2008). The Swedish version of IEQ‐S has shown satisfactory psychometric properties in this specific population (Ahlqvist Lindqvist et al. 2021).

Physical capacity was measured as dynamic balance and walking speed. Dynamic balance was measured using the 14‐item Mini‐BESTest. Each item is scored from 0, unable to perform, to 2, normal function, with a total score ranging from 0 to 28, with higher scores indicating better balance. The Mini‐BESTest has demonstrated good reliability across various populations (Meseguer‐Henarejos et al. 2025) and accurate construct validity and internal consistency in this specific population (Wagner et al. 2023). Comfortable walking speed (CWS) was measured using the 10‐m walk test (10MWT). Walking speed, expressed in metres per second (m/s), was calculated by using an average of three trials (Amatachaya et al. 2020).

### 2.2 Data Analyses

Statistical analyses were performed using IBM SPSS statistical software version 28.0.1.0 (IBM Corporation, USA). Statistical significance was set at *p* ≤ 0.05.

Descriptive statistics were calculated for all variables in relation to groups of opioid use, as well as mild and moderate to severe P‐OUD. To examine whether there were any differences between patients with mild and moderate to severe P‐OUD, the independent t‐test or Mann–Whitney *U*‐test was used for continuous variables, and the chi‐square test or the Fisher's exact test for dichotomous variables. To examine differences in pain severity, pain interference, and relief by treatment or medication between those with long‐term opioid use or P‐OUD and those without, the independent *t*‐test and the Mann–Whitney *U*‐test were used.

The bivariate association between the independent variables and long‐term opioid use and with P‐OUD was analysed using univariable binary logistic regression. The group with high‐dose long‐term opioid use stood out compared to the other groups (Figure S1). Consequently, post hoc analyses were conducted to investigate the association between the high‐dose group and the independent variables through univariable binary logistic regression analyses. All regression analyses were based on the available data. In these analyses, self‐rated health status was coded to measure the effect of a 10‐point change on the EQ VAS scale. For pain severity, the composite score of the BPI‐SF was used. For pain duration, pain < 3 years was designated as the reference category for the odds ratio. Multivariable binary logistic regression with forward selection was used to examine the multivariable association between the independent variables and long‐term opioid use and with P‐OUD. We also considered the exclusion of variables that are secondary outcome measures in the upcoming prospective studies, that is, pain severity and interference, self‐rated health and work ability, defined as employment. The multivariable analyses were conducted on patients with complete data. The model's goodness of fit was evaluated using *R* ^2^, measured by Nagelkerke as well as Cox and Snell, and also by the classification of cases (Tabachnick and Fidell 2013). When analysing the data, we allowed two models for LTOT and P‐OUD, respectively, given that they demonstrated equal fit. Reducing the number of models despite equal fit risks overlooking variables relevant to the outcomes because of collinearity (Tabachnick and Fidell 2013).

Based on the rule of thumb for logistic models, with a minimum of 5–10 events per predictor variable (EPV), the sample size exceeded the minimal requirements for the multivariable models for long‐term opioid use and P‐OUD (Vittinghoff and McCulloch 2007).

## 3 Results

**FIGURE 1:**
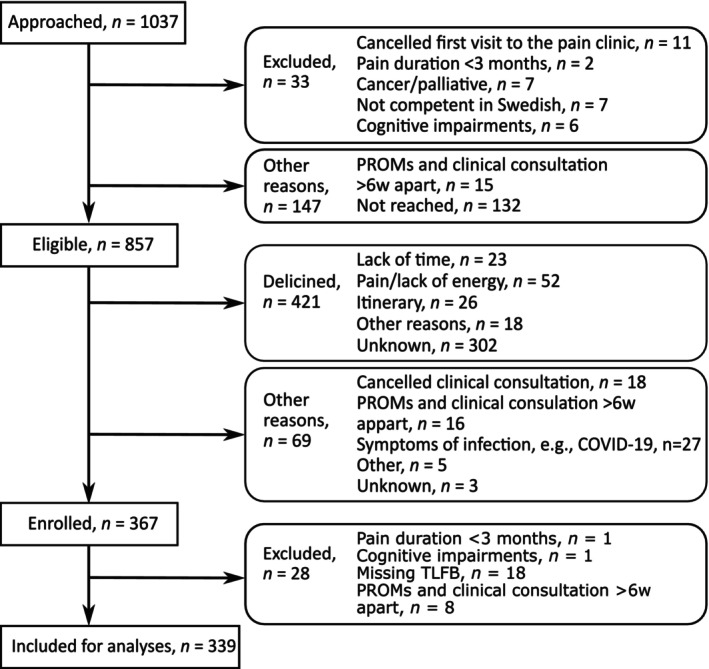
Number of approached and eligible patients, recruitment, and refusal rates.

In total, 367 participants were included in the U‐PAIN clinical cohort. Among these, 28 participants were excluded for the reasons presented in Figure 1, resulting in a final sample of 339 participants (204 females and 135 males; mean age 53.1 years, SD 16.2) for the analyses. Descriptive statistics regarding opioid use for the sample are shown in Table 1, while data related to P‐OUD can be found in Table S1. Due to incomplete responses or non‐responses, the number of participants varies for the respective independent variables indicated in the tables.

**TABLE 1:** Characteristics of all participants in relation to opioid use.

Variable^a^	All patients	No opioid use	Short or intermittent opioid use	Long‐term opioid use	
				Low to moderate doses	High doses
	*n* = 339 (100)	*n* = 145 (42.8)	*n* = 35 (10.3)	*n* = 135 (39.8)	*n* = 24 (7.1)
Prescription opioid use disorder (P‐OUD)					
P‐OUD, 12 m prevalence, *n* (%), *n* = 338					
No	295 (87.3)	139 (96.5)	31 (88.6)	110 (81.5)	15 (62.5)
Mild	28 (8.3)	2 (1.4)	3 (8.6)	17 (12.6)	6 (25.0)
Moderate	10 (3.0)	3 (2.1)	1 (2.9)	5 (3.7)	1 (4.2)
Severe	5 (1.5)	0	0	3 (2.2)	2 (8.3)
P‐OUD, lifetime prevalence, *n* (%), *n* = 338					
No	262 (77.5)	127 (88.2)	27 (77.1)	96 (71.1)	12 (50.0)
Mild	44 (13.0)	8 (5.6)	5 (14.3)	23 (17.0)	8 (33.3)
Moderate	16 (4.7)	6 (4.2)	2 (5.7)	7 (5.2)	1 (4.2)
Severe	16 (4.7)	3 (2.1)	1 (2.9)	9 (6.7)	3 (12.5)
Individual factors and demographic variables					
Female, *n* (%), *n* = 339	204 (60.2)	97 (66.9)	15 (42.9)	81 (60.0)	11 (45.8)
Age (years), mean (SD), *n* = 339	53.1 (16.2)	52.7 (16.6)	48.8 (15.3)	54.5 (16.2)	54.0 (13.7)
Born in Sweden, *n* (%), *n* = 312	270 (86.5)	116 (85.3)	27 (90.0)	109 (88.6)	18 (78.3)
Level of education, *n* (%), *n* = 312					
Elementary school (0–9 years)	75 (24.0)	33 (24.3)	5 (16.7)	30 (24.4)	7 (30.4)
High school (10–12 years)	142 (45.5)	61 (44.9)	12 (40.0)	56 (45.5)	13 (56.5)
University (> 12 years)	95 (30.4)	42 (30.9)	13 (43.3)	37 (30.1)	3 (13.0)
Employment, *n* (%), *n* = 310	105 (33.9)	53 (39.3)	15 (50.0)	35 (28.5)	2 (9.1)
Sick leave (> 25 days), *n* (%), *n* = 221	148 (67.0)	57 (63.3)	15 (60.0)	60 (69.0)	16 (84.2)
Social support, mean (SD), *n* = 304	73.5 (17.6)	74.1 (16.7)	73.5 (18.6)	74.2 (17.5)	66.9 (21.4)
Self‐rated health status^b^, mean (SD), *n* = 315	41.6 (20.6)	45.5 (20.2)	43.1 (17.8)	37.7 (20.6)	36.9 (22.8)
Substance use					
Smoking (daily), *n* (%), *n* = 312	43 (13.8)	21 (15.4)	6 (20.0)	11 (8.9)	5 (21.7)
Other nicotine use, *n* (%), *n* = 311	46 (14.8)	19 (14.0)	6 (20.0)	18 (14.6)	3 (13.6)
Hazardous alcohol use, *n* (%), *n* = 311	19 (6.1)	11 (8.1)	1 (2.9)	6 (4.9)	1 (4.3)
Illicit drug use, *n* (%), *n* = 338					
12 m prevalence	6 (1.8)	2 (1.4)	0	3 (2.2)	1 (4.2)
Lifetime prevalence	18 (5.3)	8 (5.5)	2 (5.7)	5 (3.7)	3 (12.5)
Pain characteristics					
Pain duration, *n* (%), *n* = 333					
3 months–1 year	11 (3.3)	7 (4.9)	1 (2.9)	3 (2.3)	0
> 1 year–3 years	59 (17.7)	38 (26.8)	5 (14.7)	15 (11.4)	1 (4.2)
> 3 years–10 years	100 (30.0)	42 (29.6)	12 (35.3)	40 (30.1)	6 (25.0)
> 10 years	163 (48.1)	55 (38.7)	16 (47.1)	75 (56.4)	17 (70.8)
Pain severity^c^, mean (SD), *n* = 305	6.3 (1.6)	6.2 (1.7)	5.6 (1.1)	6.5 (1.5)	6.0 (1.5)
Worst pain^d^, mean (SD), *n* = 309	7.8 (1.6)	7.5 (1.7)	8.2 (1.1)	8.0 (1.6)	7.6 (1.5)
Least pain^d^, mean (SD), *n* = 308	4.6 (2.2)	4.5 (2.3)	4.3 (1.9)	4.9 (2.2)	4.0 (2.1)
Pain on average, mean (SD), *n* = 308	6.5 (1.6)	6.2 (1.6)	7.0 (1.3)	6.6 (1.7)	6.4 (2.0)
Pain right now, mean (SD), *n* = 310	6.5 (2.0)	6.4 (2.2)	6.8 (1.9)	6.7 (2.0)	5.9 (1.8)
Pain interference^d^, mean (SD), *n* = 309	6.5 (2.2)	6.2 (2.4)	7.1 (1.9)	6.5 (2.0)	7.0 (2.0)
Relief by treatment or medication^d^, mean (SD), *n* = 304	3.3 (2.6)	2.0 (2.2)	3.2 (2.7)	4.3 (2.5)	4.9 (2.4)
Chronic pain classification, *n* (%), *n* = 332					
Primary chronic pain	148 (44.6)	67 (48.2)	16 (47.1)	56 (41.5)	9 (37.5)
Secondary chronic pain	250 (75.3)	95 (68.3)	25 (73.5)	110 (81.5)	20 (83.3)
Chronic postsurgical or posttraumatic pain	85 (25.6)	33 (23.7)	9 (26.5)	33 (24.4)	10 (41.7)
Chronic neuropathic pain	94 (28.3)	40 (28.8)	11 (32.4)	38 (28.1)	5 (20.8)
Chronic secondary musculoskeletal pain	99 (29.8)	35 (25.2)	10 (29.4)	48 (35.6)	6 (25.0)
Other secondary chronic pain conditions	25 (7.5)	3 (2.2)	3 (8.8)	15 (11.1)	4 (16.7)
More than one secondary chronic pain condition	48 (14.5)	15 (10.8)	8 (23.5)	21 (15.6)	4 (16.7)
Both primary and secondary chronic pain	66 (19.9)	23 (16.5)	7 (20.6)	31 (23.0)	5 (20.8)
Psychiatric symptoms					
Depression, mean (SD), *n* = 304	10.1 (6.5)	9.2 (6.6)	10.6 (6.4)	10.1 (6.2)	14.6 (6.2)
Anxiety, mean (SD), *n* = 307	6.5 (5.7)	5.9 (5.1)	6.6 (6.3)	6.7 (5.6)	9.3 (7.8)
ADHD, *n* (%), *n* = 306	64 (20.9)	27 (20.1)	4 (12.9)	28 (23.3)	5 (23.8)
Insomnia, mean (SD), *n* = 299	14.0 (7.2)	13.0 (7.1)	14.3 (7.2)	14.4 (7.2)	16.3 (7.9)
Traumatic life events, *n* = 313					
Number of, mean (SD)	3.1 (2.5)	2.9 (2.1)	3.1 (3.3)	3.1 (2.6)	4.5 (3.0)
Physical abuse, *n* (%)	126 (40.3)	53 (39.0)	12 (38.7)	47 (38.2)	14 (60.9)
Sexual abuse, *n* (%)	90 (28.8)	42 (30.9)	6 (19.4)	33 (26.8)	9 (39.1)
Personality, mean (SD), *n* = 314					
Neuroticism	53.2 (8.6)	53.3 (8.7)	51.7 (7.6)	53.5 (8.5)	53.2 (9.3)
Extraversion	49.0 (7.1)	48.8 (7.0)	49.1 (8.0)	49.2 (6.8)	49.6 (8.5)
Aggressiveness	50.6 (5.9)	50.8 (6.0)	49.9 (6.9)	50.4 (5.4)	50.9 (6.7)
Substance use disorder^e^, *n* (%), *n* = 338					
12 m prevalence	12 (3.6)	6 (4.2)	1 (2.9)	5 (3.7)	0
Lifetime prevalence	32 (9.5)	12 (8.3)	4 (11.4)	13 (9.6)	3 (12.5)
Cognitive‐behavioural variables					
Pain Catastrophising, mean (SD), *n* = 303	24.0 (12.7)	22.1 (12.7)	24.9 (12.7)	24.9 (12.2)	29.4 (14.2)
Fear of movement/(re) injury, mean (SD), *n* = 297	24.0 (7.6)	23.5 (7.9)	23.6 (6.8)	23.9 (7.4)	27.3 (8.1)
Pain self‐efficacy, mean (SD), *n* = 307	6.0 (3.5)	6.6 (3.5)	6.2 (3.3)	5.7 (3.4)	4.0 (3.6)
Injustice, mean (SD), *n* = 303	22.8 (12.6)	21.2 (12.8)	23.8 (12.3)	22.9 (12.0)	30.2 (12.9)
Physical capacity					
Balance, mean (SD), *n* = 323	20.0 (6.4)	20.7 (6.0)	20.6 (6.0)	19.2 (6.8)	18.6 (7.1)
Walking speed (m/s), mean (SD), *n* = 311	1.1 (0.3)	1.1 (0.3)	1.1 (0.3)	1.1 (0.3)	0.9 (0.3)

^a^ Differences in the n for each independent variable are due to incomplete responses or non‐response.

^b^ Self‐rated health status measured by EQ VAS 0–100.

^c^ Composite score, that is, the mean of the worst pain, least pain, pain on average and pain right now.

^d^ The past 24 h.

^e^ OUD was not included in the SUD.

In the sample, 194 patients (57%) were using opioids, and 159 patients (47%) were classified as long‐term opioid users. Oxycodone was the most commonly prescribed opioid analgesic across all groups: for other opioids, see Table 2. Among long‐term opioid users, 23 patients (14%) exhibited mild P‐OUD, while 11 patients (7%) fulfilled the criteria for moderate or severe P‐OUD. There were no differences in pain severity or pain interference between long‐term opioid users and non‐long‐term opioid users, nor between those with and without P‐OUD. However, individuals with long‐term opioid use or P‐OUD reported significantly higher levels of relief from treatment or medication compared to those without these conditions (Figure S2).

**TABLE 2:** The most commonly prescribed opioids in the sample.

Type of opioid used the last 30 days	All opioid users	Opioid use			P‐OUD, 12 m prevalence		
		Short or intermittent	Long‐term		No	Mild	Moderate or severe
			Low to moderate doses	High doses			
	*n* (%)	*n* (%)	*n* (%)	*n* (%)	*n* (%)	*n* (%)	*n* (%)
	194 (100)	35 (18.0)	135 (69.6)	24 (12.4)	156 (80.4)	26 (13.4)	12 (6.2)
Oxycodone	50 (25.8)	10 (28.6)	33 (24.4)	7 (29.2)	42 (26.9)	4 (15.4)	4 (33.3)
Tramadol	26 (13.4)	5 (14.3)	17 (12.6)	4 (16.7)	20 (12.8)	4 (15.4)	2 (16.7)
Codeine and paracetamol	18 (9.3)	6 (17.1)	12 (8.9)	0	16 (10.3)	2 (7.7)	0
Buprenorphine	17 (8.8)	5 (14.3)	12 (8.9)	0	15 (9.6)	2 (7.7)	0
Other opioids	19 (9.8)	2 (20.0)	8 (5.9)	9 (37.5)	13 (8.3)	5 (19.2)	1 (8.3)
More than one opioid^a^	64 (33.0)	7 (20.0)	53 (39.3)	4 (16.7)	50 (32.1)	9 (34.6)	5 (41.7)

^a^ Oxycodone was the most common opioid among patients with more than one opioid (*n* = 40, 62.5%).

### 3.1 Associations Between Patient Characteristics and Long‐Term Opioid Use

In the univariable regression analyses, pain duration and pain catastrophising were positively associated with long‐term opioid use. Conversely, employment status, self‐rated health, pain self‐efficacy, balance, and walking speed were negatively associated with long‐term opioid use (Table 3).

**TABLE 3:** Biopsychosocial variables associated with long‐term opioid use, and with prescription opioid use disorder (P‐OUD). Univariable analyses.

Variable	Univariable binary regression analyses								
	Long‐term opioid use						P‐OUD, 12 m prevalence		
	Low, moderate, and high doses			High doses					
	OR^a^	95% CI^b^	*p* ^c^	OR^a^	95% CI^b^	*p* ^c^	OR^a^	95% CI^b^	*p* ^c^
Prescription opioid use									
Long‐term opioid use							5.14	2.38–11.10	**< 0.001**
Individual factors and demographic variables									
Female sex	0.83	0.54–1.29	0.413	0.53	0.23–1.23	0.142	0.73	0.39–1.40	0.348
Age (years)	1.01	1.00–1.02	0.154	1.01	0.98–1.03	0.791	0.97	0.95–0.99	**0.011**
Level of education^d^									
High school (10–12 years)	0.97	0.55–1.70	0.917	0.98	0.37–2.57	0.966	0.95	0.42–2.18	0.906
University (> 12 years)	0.75	0.41–1.37	0.348	0.32	0.08–1.27	0.105	1.12	0.47–2.69	0.794
Employment, *n*/years	0.49	0.30–0.79	**0.004**	0.18	0.04–0.78	**0.022**	1.09	0.55–2.16	0.799
Social support	1.00	0.98–1.01	0.656	1.40	0.39–4.99	0.600	1.00	0.98–1.02	0.822
Self‐rated health status^e^	0.83	0.74–0.93	**0.001**	0.88	0.71–1.10	0.267	0.86	0.72–1.01	0.069
Substance use									
Smoking (daily), *n*/years	0.63	0.33–1.23	0.177	1.83	0.64–5.23	0.256	2.63	1.20–5.74	**0.015**
Other nicotine use, *n*/years	0.96	0.51–1.79	0.886	0.90	0.26–3.18	0.874	1.76	0.78–3.99	0.173
Hazardous alcohol use, *n*/years	0.64	0.25–1.68	0.366	0.68	0.09–5.35	0.716	1.21	0.34–4.35	0.769
Illicit drug use, lifetime, *n*/years	0.90	0.34–2.33	0.821	2.85	0.76–10.62	0.119	1.40	0.39–5.05	0.607
Pain characteristics									
Pain duration^f^									
> 3 years–10 years	2.29	1.18–4.41	**0.014**	4.40	0.52–37.42	0.174	1.32	0.46–3.75	0.604
> 10 years	3.48	1.89–6.40	**< 0.001**	8.03	1.05–61.60	**0.045**	1.95	0.76–5.00	0.165
Pain severity	1.08	0.94–1.25	0.278	1.08	0.94–1.25	0.278	1.04	0.85–1.28	0.687
Pain interference	1.06	0.95–1.17	0.302	1.06	0.95–1.17	0.302	1.02	0.88–1.19	0.794
Psychiatric symptoms									
Depression	1.03	1.00–1.07	0.078	1.12	1.05–1.20	**< 0.001**	1.08	1.03–1.13	**0.003**
Anxiety	1.03	0.99–1.08	0.097	1.09	1.02–1.17	**0.016**	1.09	1.03–1.15	**0.001**
ADHD	1.32	0.76–2.29	0.323	1.12	0.42–3.40	0.736	1.62	0.76–3.46	0.216
Insomnia	1.03	1.00–1.06	0.098	1.05	0.98–1.12	0.132	1.05	1.00–1.10	0.054
Traumatic life events									
Number of	1.07	0.98–1.16	0.159	1.22	1.05–1.42	**0.008**	1.27	1.12–1.44	**< 0.001**
Physical abuse, *n*/years	1.13	0.72–1.77	0.607	2.47	1.04–5.90	**0.041**	2.08	1.07–4.05	**0.030**
Sexual abuse, *n*/years	1.00	0.61–1.64	0.996	1.66	0.69–3.98	0.257	1.70	0.86–3.37	0.126
Personality									
Neuroticism	1.01	0.98–1.03	0.652	1.00	0.95–1.05	0.986	1.04	1.00–1.08	**0.033**
Extraversion	1.01	0.98–1.04	0.621	1.01	0.95–1.08	0.680	1.07	1.02–1.12	**0.007**
Aggressiveness	1.00	0.96–1.03	0.997	1.01	0.94–1.09	0.774	1.03	0.97–1.09	0.324
Substance use disorder, lifetime, *n*/years	1.14	0.55–2.36	0.725	1.40	0.39–4.99	0.600	3.13	1.34–7.32	**0.008**
Cognitive‐behavioural variables									
Pain catastrophising	1.02	1.00–1.04	**0.047**	1.04	1.00–1.07	**0.040**	1.04	1.01–1.07	**0.003**
Fear of movement/(re) injury	1.02	0.99–1.05	0.301	1.06	1.00–1.13	**0.032**	1.07	1.03–1.12	**0.001**
Pain self‐efficacy	0.91	0.86–0.98	**0.007**	0.83	0.72–0.95	**0.005**	0.99	0.90–1.09	0.830
Injustice	1.02	1.00–1.03	0.105	1.05	1.02–1.09	**0.005**	1.05	1.02–1.08	**< 0.001**
Physical capacity									
Balance	0.96	0.93–1.00	**0.035**	0.97	0.91–1.03	0.291	1.03	0.97–1.09	0.363
Walking speed	0.47	0.22–0.98	**0.045**	0.17	0.04–0.70	**0.013**	1.56	0.51–4.79	0.438

*Note:* The grey highlights significant variables.

^a^ Odds ratio.

^b^ 95% confidence interval.

^c^ *p*‐value. Significant values are presented in bold.

^d^ Elementary school (0–9 years) was the reference category for the OR.

^e^ Self‐rated health status was coded so that each 1 unit change on the scale represents a 10‐step change on the EQ VAS 0–100 scale.

^f^ Pain ≤ 3 years was the reference category for the odds ratio.

Variables associated with long‐term use at high doses partially overlapped with those associated with long‐term use regardless of dose. However, high‐dose use was associated with a greater number of reported psychiatric and behavioural issues (Table 3).

In the multivariable regression analyses, two alternative models were considered relevant, as shown in Table 4. In model 1, variables associated with long‐term opioid use were pain duration, employment, and pain catastrophising. When employment was excluded, model 2 revealed that pain duration, self‐rated health, and balance were associated with long‐term opioid use. This illustrates how different combinations of variables accounted for variation in long‐term opioid use, that is, either having a long pain duration, combined with high pain catastrophising and unemployment, or combined with a low self‐rated health and poor balance, were associated with long‐term opioid use.

**TABLE 4:** Variables associated with long‐term opioid use. Multivariable analyses.

Variable	Multivariable regression analyses					
	Long‐term opioid use					
	Model 1			Model 2		
	*n* = 234			*n* = 234		
	OR^a^	95% CI^b^	*p* ^c^	OR^a^	95% CI^b^	*p* ^c^
Pain duration^e^						
> 3 years–10 years	3.16	1.34–7.43	0.008	3.02	1.28–7.14	0.012
> 10 years	5.33	2.44–11.6	< 0.001	5.07	2.33–11.02	< 0.001
Employment, years/*n*	0.51	0.28–0.91	0.024			
Pain catastrophising	1.02	1.00–1.05	0.049			
Self‐rated health status^d^				0.85	0.74–0.98	0.027
Balance				0.95	0.93–1.00	0.049

^a^ Odds ratio.

^b^ 95% confidence interval.

^c^ *p*‐value.

^d^ Self‐rated health status was coded so that each 1 unit change on the scale represents a 10‐step change on the EQ VAS 0–100 scale.

^e^ Pain ≤ 3 years was the reference category for the odds ratio.

### 3.2 Associations Between Patient Characteristics and Prescription Opioid Use Disorder

In the univariable analyses, long‐term opioid use, smoking, SUD, anxiety, depression, neuroticism, extraversion, fear of movement or (re)injury, and pain catastrophising were positively associated with P‐OUD. Age was negatively associated with P‐OUD (Table 3).

For the 12‐month prevalence of P‐OUD, several different multivariable models were possible, as presented in Table 5. Due to the high association between perceived injustice and pain catastrophising, both variables could not be included in the model simultaneously. This resulted in two different models that explain the variation in the dependent variable. In Model 1, without perceived injustice, variables associated with P‐OUD were long‐term opioid use, age, number of traumas, and pain catastrophising. In Model 2, where perceived injustice replaced pain catastrophising, the variables associated with P‐OUD were long‐term opioid use, age, number of traumas, and fear of movement. Different combinations of variables explained the variation in P‐OUD, indicating that long‐term opioid use, lower age, and higher exposure to trauma, combined with either high pain catastrophising or high fear of movement, were associated with P‐OUD.

**TABLE 5:** Variables associated with prescription opioid use disorder in the multivariable analyses.

Variable	Multivariable regression analyses					
	Prescription opioid use disorder, 12 m prevalence					
	Model 1			Model 2		
	*n* = 234			*n* = 234		
	OR^a^	95% CI^b^	*p* ^c^	OR^a^	95% CI^b^	*p* ^c^
Long‐term opioid use	8.65	2.95–25.36	< 0.001	9.21	3.12–27.17	< 0.001
Age (years)	0.96	0.94–0.99	0.017	0.97	0.94–0.99	0.019
Number of traumatic life events	1.24	1.06–1.44	0.006	1.23	1.06–1.44	0.007
Pain catastrophising	1.04	1.01–1.08	0.025			
Fear of movement/(re) injury				1.07	1.01–1.14	0.028

^a^ Odds ratio.

^b^ 95% confidence interval.

^c^ *p*‐value.

## 4 Discussion

The U‐PAIN cohort provides comprehensive data on patients referred to specialised pain care. This unique characterisation of biological, psychological and social factors enabled an examination of how these characteristics were associated with long‐term opioid use and P‐OUD. Our sample had a relatively high proportion of opioid use compared to other clinical samples (De Sola et al. 2020; Grelz et al. 2022). Hence, the results contribute to the body of knowledge on what factors are critical to assess and address for managing chronic pain when considering LTOT in specialised care.

The likelihood of long‐term opioid use increased with pain duration, unemployment, increased pain catastrophising, lower self‐rated health and worse balance. This may reflect how psychological and social factors interact with neurobiological mechanisms in the development of chronic pain (Borsook et al. 2018). Persistent pain is a stressor that reinforces emotional distress and pain‐related disability through pain‐conditioned cues and heightened sensitivity salience to pain and pain‐related stimuli (Elman and Borsook 2016). Regarding opioid use, similar mechanisms that drive pain chronicity and disability may impact the likelihood of prolonged opioid use through pain‐related behaviours and an overreliance on opioids. This could result from neuroadaptations leading to a reward deficiency, pain sensitisation, and aberrant learning that drive continued opioid use (Borsook et al. 2018). Thus, a vicious cycle of pain beliefs, negative affect, and avoidance, as described in the fear‐avoidance model (Edwards et al. 2016; Vlaeyen et al. 2016), maintains opioid therapy due to fear of increased pain. Furthermore, patients on long‐term high opioid doses reported more psychiatric symptoms, pain catastrophising, fear of movement and injustice, and lower pain self‐efficacy than patients with no or low to moderate opioid doses, similar to those who reported P‐OUD. This suggests that even if a patient's opioid use does not meet the DSM‐5 criteria for P‐OUD, high‐dose LTOT can still be problematic and have adverse effects that need attention. This aligns with how long‐term exposure to opioids, especially at high doses, affects the ability to feel pleasure, socialise, and prioritise due to tolerance and withdrawal, leading to structural changes in the brain's emotional and motivational systems, regardless of P‐OUD (Ballantyne et al. 2019). This similarity between high‐dose opioid users and those reporting P‐OUD evokes questions regarding how P‐OUD differs from opioid dependence in this population and what constructs are most important to measure. We used a structured interview to assess the DSM‐5 criteria for P‐OUD to define problematic opioid use. The DSM‐5 criteria adjusted for chronic pain, as described by Hasin et al. (2022), provide a moderate estimate of problematic opioid use in patients with LTOT. This is because these patients often attribute their decreased physical and psychological well‐being, as well as their decreased ability to fulfil social and relational obligations, to pain‐related ailments rather than their opioid use (Manhapra et al. 2020). Furthermore, patients with LTOT seldom take more opioids than prescribed and report increased pain as the driver of opioid use rather than craving or preoccupation with the drug. This can make it hard to capture P‐OUD using self‐reported data, even when collected through a structured interview, and it may also fail to detect clinically relevant problematic opioid use in this particular population. Given the known adverse effects that tolerance and withdrawal have on chronic pain conditions, it is questionable to apply conditional exclusion, that is, opioid use under medical supervision, for these criteria (Ballantyne et al. 2019). There are ongoing discussions on how to best define and measure problematic opioid use in patients with chronic pain and LTOT, and this is a field that needs further research (Manhapra et al. 2020).

Patients with long‐term opioid use and those with P‐OUD reported better therapeutic effects from their treatment than those not prescribed opioids. This is a known argument from both patients and prescribers for continued LTOT (Brooks et al. 2015; Ljungvall et al. 2020; Ljungvall, Öster, et al. 2022). However, there were no differences in self‐reported pain severity or pain interference between opioid users and non‐users. This illustrates the clinical dilemma associated with LTOT, that is, patients report improvement but do not report less pain or pain interference on average than patients who do not receive opioids. Although our study design does not allow for causal inference regarding opioid use and pain relief, it can be hypothesised that the reported relief reflects alleviation of withdrawal symptoms rather than effective treatment of underlying pain (Ballantyne et al. 2019). Furthermore, the measure of pain severity captures an aggregated pain experience involving sensory and emotional aspects, that is, suffering, where opioids affect all of these aspects (Borsook et al. 2018).

Our results indicate that patients with particular primary and neuropathic chronic pain conditions received LTOT despite opioids being discouraged in such circumstances (Dowell et al. 2022). There also seems to be an adverse selection of patients receiving LTOT, characterised by longer pain duration and higher rates of co‐morbidity, which in turn elevate the risk for P‐OUD (Sullivan and Howe 2013). In this study, pain catastrophising, fear of movement, younger age, the number of experienced traumas, and long‐term opioid use increased the likelihood for P‐OUD. These are well‐known risk factors for OUD (Martel et al. 2013; Verner et al. 2023; Webster 2017). Our results reflect the importance of applying the biopsychosocial model when assessing risk factors and considering multimodal pain treatment before initiating opioid therapy. For those who have already developed P‐OUD, it is important to offer integrated treatment targeting both pain management and P‐OUD (Oldfield et al. 2018). Thus, our study further highlights the importance of providing treatment that disrupts the fear avoidance circle for patients with LTOT, regardless of whether they suffer from P‐OUD or not (Linton 2013; Sullivan et al. 2011).

### 4.1 Strengths and Limitations

An important strength of our study is the clinical sample, which includes patients with and without long‐term opioid use. The thorough biopsychosocial assessment has enabled a comprehensive characterisation regarding both long‐term opioid use and P‐OUD, which is rarely seen in the literature. Self‐reports were used for assessing opioid use and P‐OUD, introducing a risk of recall bias regarding actual substance use and substance‐related behaviour. Nevertheless, the reliability of the current self‐reports was acceptable, as reported in the feasibility study of the U‐PAIN cohort (Ljungvall, Lind, et al. 2022). Self‐reported data allow for detecting patterns of opioid use that cannot be identified when using prescription register data (Karmali et al. 2020). Moreover, the 12‐month prevalence of P‐OUD in our study was similar to what has previously been reported in chronic pain samples with LTOT (Vowles et al. 2015).

This study was conducted amidst the COVID‐19 pandemic, resulting in a halt in new recruitments for a period, thereby impeding our ability to reach the intended number of participants. The low participation rate and the internal attrition are important limitations that need to be addressed, as they increase the risk of systematic bias. Declining participation or non‐completion of the measures may be due to lack of time, pain disability, or the extensive amount of measures. Moreover, the high proportion of patients with prescribed opioids could be related to selection bias, as patients with LTOT were more prone to participate given that the overall aim of the U‐PAIN cohort study is to examine the benefits and risks associated with long‐term opioid therapy. Given the size of our data set, the risk of missing major patterns is small, and since our focus is on associations identified through multivariable regression analyses, the potential exclusion of certain subgroups is less likely to bias our findings. Also, our results are consistent with previous research regarding factors associated with LTOT and P‐OUD in clinical chronic pain populations (Campbell et al. 2020; De Sola et al. 2020; Grelz et al. 2022; Vowles et al. 2015), which supports that our sample well represents the target population.

Some measures lack full validation in this population, possibly affecting reliability, though all showed acceptable consistency in the U‐PAIN feasibility study (Ljungvall, Lind, et al. 2022).

Finally, the cross‐sectional design does not allow us to establish causality or predictors of either long‐term opioid use or P‐OUD. As described above, the interactions between opioid use and pain are complex, and one condition can exacerbate the other, that is, these associations are often bidirectional. Hence, we chose to include our prospective secondary outcome measures in the models for the cross‐sectional data, although they will be regarded as dependent variables in the prospective studies.

## 5 Conclusions, Clinical Implications, and Future Research

Our results demonstrate that pain duration, unemployment, increased pain catastrophising, lower self‐rated health, and limitations in balance increased the likelihood of long‐term opioid use. Long‐term opioid use, younger age, number of traumatic life events, increased pain catastrophising, and fear of movement increased the likelihood of suffering from P‐OUD. Patients with long‐term use combined with high doses of opioids had similar characteristics and reported more disability than patients with no opioid use or long‐term use of low‐ to moderate opioid doses. Our results demonstrate the importance of identifying these factors before initiating and during LTOT.

Today, patients undergoing opioid therapy are often excluded from interdisciplinary and multimodal rehabilitation. Additionally, many of the variables associated with LTOT and P‐OUD can be targeted within interdisciplinary pain rehabilitation. This requires pain rehabilitation teams to also have competency in opioid dependence and substance use disorders.

Future research will include prospective studies to confirm the value of the prediction models for adverse effects such as P‐OUD relating to LTOT in patients with chronic pain. Moreover, these studies will aim to identify whether patients with certain characteristics benefit from LTOT.

## Author Contributions

All authors have made substantial contributions to the article and take public responsibility for appropriate portions of the content. P. Åsenlöf was the principal investigator of the study, and S. Wagner, H. Ljungvall, H. Zetterberg, R. Karlsten, and L. Ekselius made contributions to its design. S. Wagner, H. Ljungvall, H. Zetterberg, and R. Karlsten contributed to the data collection. S. Wagner performed the analysis, and all authors contributed to the interpretation of the data. S. Wagner and H. Ljungvall drafted the manuscript. All authors discussed the results, commented on the manuscript, and gave approval for the final version to be published.

## Conflicts of Interest

H.L. has received honoraria from Nordic Drugs AB and Indivior for presenting educational seminars related to person‐centred care in addiction treatment and on chronic pain and opioid therapy. The remaining authors declare no conflicts of interest.

## Supporting information


Figure S1.

Figure S2.

Table S1.
