# Supplementary material for: Opioid Use and Prescription Opioid Use Disorder: Biopsychosocial Characterisation of a Clinical Chronic Pain Cohort
Source: Eur J Pain. 2025 Jul 19;29(7):e70081. doi: 10.1002/ejp.70081 (PMC12275012; doi:10.1002/ejp.70081)
Supplement: Supplementary file 1 — Figure S1. [file EJP-29-0-s002.docx]

**Supplementary Information for**

**Opioid Use and Prescription Opioid Use Disorder: Biopsychosocial characterisation of a Clinical Chronic Pain Cohort**

Sofia Wagner^1,2^, Hanna Ljungvall^1^, Hedvig Zetterberg^1^, Rolf Karlsten^2,3^, Lisa Ekselius^4^, Pernilla Åsenlöf^1,2^


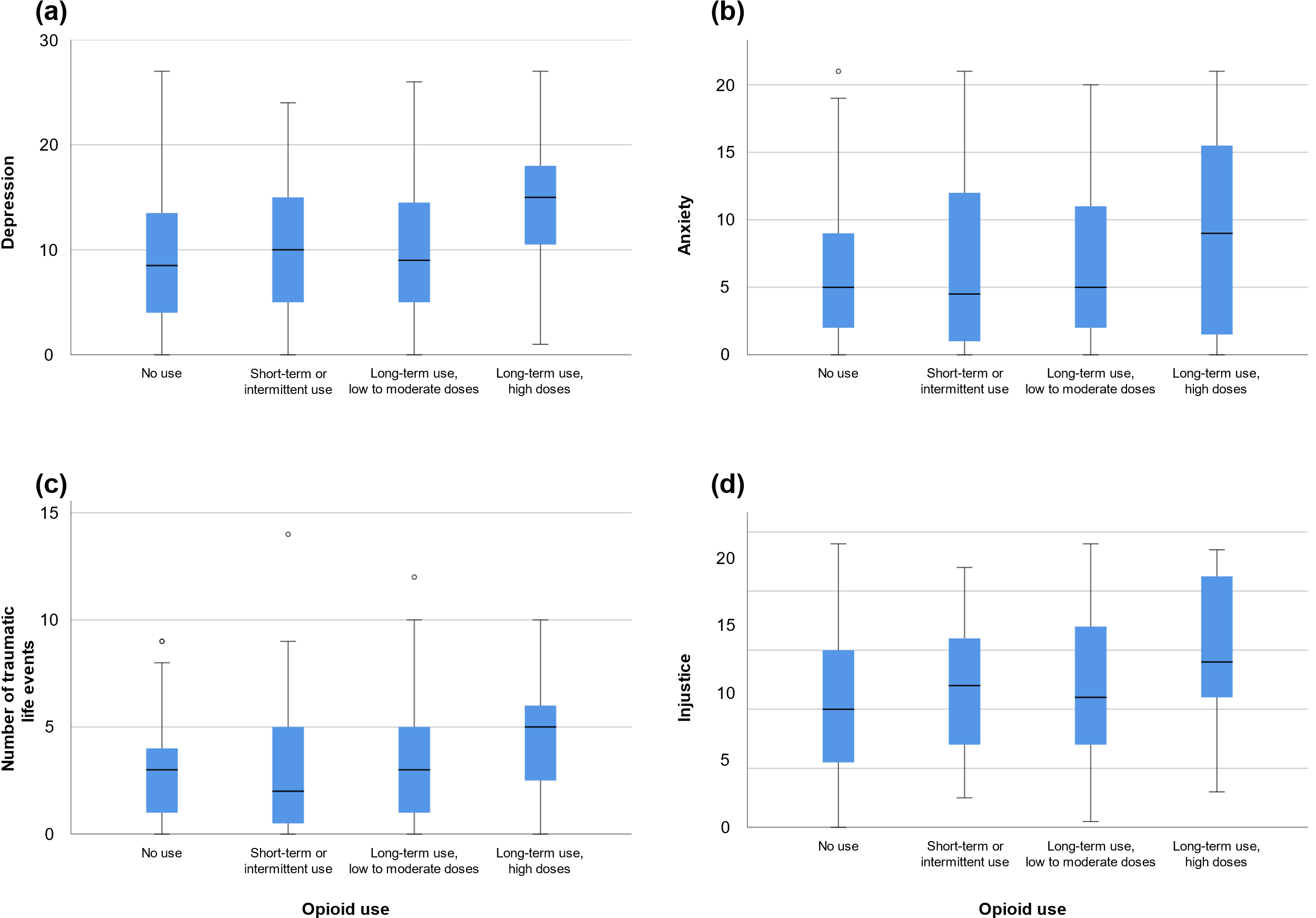


**Figure S1** Boxplots illustrating differences in depression (A), anxiety (B), number of traumatic life events (C), and injustice (D) between high-dose long-term opioid users and the other opioid use groups.
