# Supplementary material for: Opioid Use and Prescription Opioid Use Disorder: Biopsychosocial Characterisation of a Clinical Chronic Pain Cohort
Source: Eur J Pain. 2025 Jul 19;29(7):e70081. doi: 10.1002/ejp.70081 (PMC12275012; doi:10.1002/ejp.70081)
Supplement: Supplementary file 2 — Figure S2. [file EJP-29-0-s003.docx]

**Supplementary Information for**

**Opioid Use and Prescription Opioid Use Disorder: Biopsychosocial Characterisation of a Clinical Chronic Pain Cohort**

Sofia Wagner^1,2^, Hanna Ljungvall^1^, Hedvig Zetterberg^1^, Rolf Karlsten^2,3^, Lisa Ekselius^4^, Pernilla Åsenlöf^1,2^

^
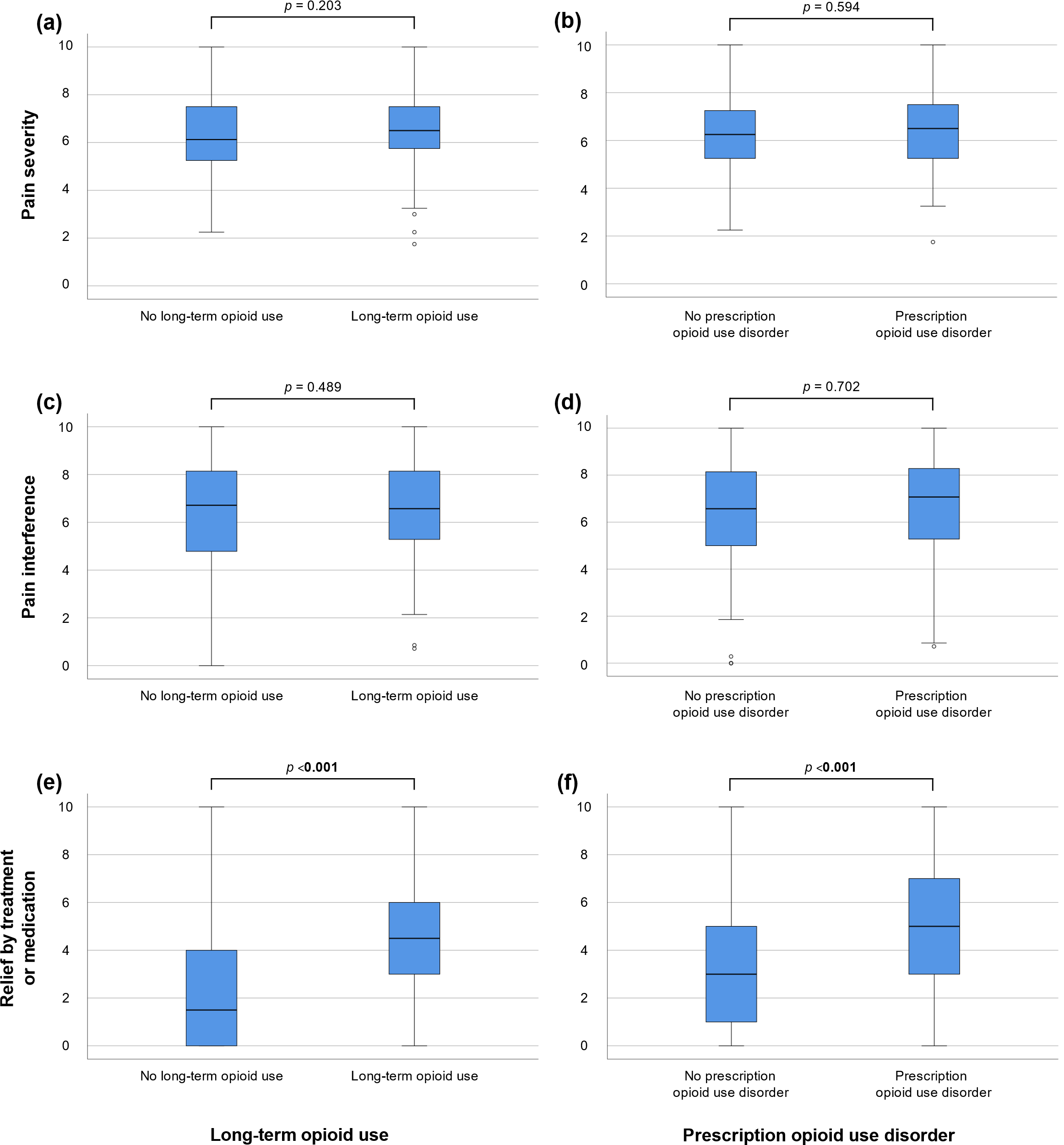
^

**Figure S2** Difference in pain characteristics between individuals with and without long-term opioid use and with and without prescription opioid use disorder (P-OUD).

*Differences between individuals with or without long-term opioid use (a, c, e) and with and without P-OUD (b, d, f) were examined with the student t-test or Mann-Whitney U test. Significant *p* values are presented in bold. There were no differences in pain severity (*U* = 12559, *z* = 1.273, *p* = .203) or pain interference (*U* = 12441, *z* = 0.691, *p* = .489) between individuals with and without long-term opioid use. Neither in pain severity (*U* = 5783, *z* = 0.533, *p* = .594) or pain interference (*U* = 5791, *z* = 0.383, *p* = .702) between individuals with and without P-OUD. However, differences in relief from treatment or medication were found for both long-term opioid users (t(302) = -7.78, p <.001) and those with P-OUD (t(301) = -4.05, p <.001) compared with those without.
