# Supplementary material for: Opioid Use and Prescription Opioid Use Disorder: Biopsychosocial Characterisation of a Clinical Chronic Pain Cohort
Source: Eur J Pain. 2025 Jul 19;29(7):e70081. doi: 10.1002/ejp.70081 (PMC12275012; doi:10.1002/ejp.70081)
Supplement: Supplementary file 3 — Table S1. [file EJP-29-0-s001.docx]

**Supplementary Information for**

**Opioid Use and Prescription Opioid Use Disorder: Biopsychosocial Characterisation of a Clinical Chronic Pain Cohort**

Sofia Wagner^1,2^, Hanna Ljungvall^1^, Hedvig Zetterberg^1^, Rolf Karlsten^2,3^, Lisa Ekselius^4^, Pernilla Åsenlöf^1,2^

**TableS1** Characteristics of all participants in relation to prescription opioid use disorder (P-OUD).

| **Variable^a^** | **All patients**  ***n* = 338 (100)** | **No**  **P-OUD**  ***n* = 295 (87.0)** | **P-OUD,**  **12 m prevalence**  ***n* = 43**  **(12.7)** | **P-OUD, 12 m prevalence** | | |
| --- | --- | --- | --- | --- | --- | --- |
|  |  |  |  | **Mild**  ***n* = 28**  **(8.3)** | **Moderate or severe**  ***n* = 15**  **(4.4)** | ***p***  **value^b^** |
| **OPIOID USE** |  |  |  |  |  |  |
| Long-term opioid use, *n* (%), *n* = 339 |  |  |  |  |  | .696 |
| No | 179 (53.0) | 170 (57.6) | 9 (20.9) | 5 (17.9) | 4 (26.7) |  |
| Yes | 159 (47.0) | 125 (42.4) | 34 (79.1) | 23 (82.1) | 11 (73.3) |  |
| **INDIVIDUAL FACTORS AND DEMOGRAPHIC VARIABLES** |  |  |  |  |  |  |
| Female, *n* (%), *n* = 338 | 203 (60.1) | 180 (61.0) | 23 (53.5) | 16 (57.1) | 7 (46.7) | .512 |
| Age (y), mean (SD), *n* = 338 | 53.2 (16.1) | 54.0 (16.4) | 47.2 (12.7) | 48.8 (13.0) | 44.3 (11.7) | .266 |
| Born in Sweden, *n* (%), *n* = 311 | 269 (86.5) | 234 (87.0) | 35 (83.3) | 23 (85.2) | 12 (80.0) | .686 |
| Level of education, *n* (%), *n* = 311 |  |  |  |  |  | .175 |
| Elementary school (0–9 years) | 75 (24.1) | 65 (24.2) | 10 (23.8) | 9 (33.3) | 1 (6.7) |  |
| High school (10–12 years) | 141 (45.3) | 123 (45.7) | 18 (42.9) | 10 (37.0) | 8 (53.3) |  |
| University (> 12 years) | 95 (30.5) | 81 (30.1) | 14 (33.3) | 8 (29.6) | 6 (40.0) |  |
| Employment, *n* (%), *n* = 309 | 105 (34.0) | 90 (33.7) | 15 (35.7) | 7 (25.9) | 8 (53.3) | .076 |
| Sick leave (> 25 days), *n* (%), *n* = 220 | 148 (67.3) | 124 (67.4) | 24 (66.7) | 16 (72.7) | 8 (57.1) | .471 |
| Social support, mean (SD), *n* = 303 | 73.5 (17.7) | 73.6 (17.6) | 72.9 (18.3) | 73.0 (19.3) | 72.8 (17.0) | .968 |
| Self-rated health status^c^, mean (SD), *n* = 314 | 41.5 (20.6) | 42.4 (20.7) | 36.0 (19.5) | 33.5 (20.2) | 40.9 (17.5) | .253 |
| **SUBSTANCE USE** |  |  |  |  |  |  |
| Smoking (daily), *n* (%), *n* = 311 | 43 (13.8) | 32 (11.9) | 11 (26.2) | 6 (22.2) | 5 (33.3) | .481 |
| Other nicotine use, *n* (%), *n* = 310 | 46 (14.8) | 37 (13.8) | 9 (22.0) | 5 (19.2) | 4 (26.7) | .701 |
| Hazardous alcohol use, *n* (%), *n* = 310 | 19 (6.1) | 16 (6.0) | 3 (7.1) | 3 (11.1) | 0 (0) | .541 |
| Illicit drug use, *n* (%), *n* = 338 |  |  |  |  |  |  |
| 12 m prevalence | 6 (1.8) | 5 (1.7) | 1 (2.3) | 1 (3.6) | 0 | 1.00 |
| Life time prevalence | 18 (5.3) | 15 (5.1) | 3 (7.0) | 3 (10.7) | 0 | .541 |
| **PAIN CHARACTERISTICS** |  |  |  |  |  |  |
| Pain duration, *n* (%), *n* = 332 |  |  |  |  |  | 1.00 |
| 3 months–1 year | 11 (3.3) | 11 (3.8) | 0 (0) | 0 (0) | 0 (0) |  |
| > 1 year–3 years | 59 (17.8) | 53 (18.3) | 6 (14.3) | 4 (14.8) | 2 (13.3) |  |
| > 3 years–10 years | 100 (30.1) | 89 (30.7) | 11 (26.2) | 16 (25.9) | 4 (26.7) |  |
| > 10 years | 162 (48.8) | 137 (47.2) | 25 (59.5) | 27 (59.3) | 9 (60.0) |  |
| Pain severity^d^, mean (SD), *n* = 304 | 6.3 (1.6) | 6.3 (1.6) | 6.4 (1.5) | 6.5 (1.7) | 6.3 (2.0) | .841 |
| Worst pain^e^, mean (SD), *n* = 308 | 7.8 (1.6) | 7.8 (1.6) | 8.0 (1.9) | 8.2 (1.9) | 7.7 (1.9) | .462 |
| Least pain^e^, mean (SD), *n* = 307 | 4.6 (2.2) | 4.6 (2.2) | 4.7 (2.3) | 4.6 (2.4) | 4.7 (2.3) | .893 |
| Pain on average, mean (SD), *n* = 307 | 6.5 (1.7) | 6.4 (1.6) | 6.6 (2.1) | 6.5 (2.1) | 6.7 (2.1) | .757 |
| Pain right now, mean (SD), *n* = 309 | 6.5 (2.0) | 6.5 (2.0) | 6.4 (2.2) | 6.7 (1.8) | 5.8 (2.7) | .192 |
| Pain interference^e^, mean (SD), *n* = 308 | 6.5 (2.2) | 6.5 (2.2) | 6.6 (2.2) | 6.8 (1.8) | 6.1 (2.7) | .355 |
| Relief by treatment or medication^e^, mean (SD), *n* = 303 | 3.3 (2.6) | 3.0 (2.6) | 4.8 (2.5) | 4.5 (2.5) | 5.3 (2.5) | .298 |
| Chronic pain classification, *n* (%), *n* = 331 |  |  |  |  |  |  |
| Primary chronic pain | 147 (44.4) | 129 (44.8) | 18 (41.9) | 11 (39.3) | 7 (46.7) | .857 |
| Secondary chronic pain | 250 (75.5) | 213 (74.0) | 37 (86.0) | 24 (85.7) | 13 (86.7) | .538 |
| Chronic postsurgical or posttraumatic pain | 85 (25.7) | 70 (24.3) | 15 (34.9) | 11 (39.3) | 4 (26.7) | 1.00 |
| Chronic neuropathic pain | 94 (28.4) | 85 (29.5) | 9 (20.9) | 5 (17.9) | 4 (26.7) | 1.00 |
| Chronic secondary musculoskeletal pain | 99 (29.9) | 85 (29.5) | 14 (32.6) | 10 (35.7) | 4 (26.7) | 1.00 |
| Other secondary chronic pain conditions | 25 (7.6) | 18 (6.3) | 7 (16.3) | 5 (17.9) | 2 (13.3) | .315 |
| More than one secondary chronic pain condition | 48 (14.5) | 41 (14.2) | 7 (16.3) | 6 (21.4) | 1 (6.7) | .438 |
| Both primary and secondary chronic pain | 66 (19.9) | 54 (18.8) | 12 (27.9) | 7 (25.0) | 5 (33.3) | .191 |
| **PSYCHIATRIC SYMPTOMS** |  |  |  |  |  |  |
| Depression, mean (SD), *n* = 303 | 10.1 (6.5) | 9.6 (6.3) | 12.9 (7.0) | 11.8 (6.0) | 15.0 (8.4) | .155 |
| Anxiety, mean (SD), *n* = 306 | 6.5 (5.7) | 6.1 (5.4) | 9.2 (6.6) | 8.7 (6.4) | 10.1 (7.0) | .507 |
| ADHD, *n* (%), *n* = 305 | 63 (20.7) | 52 (19.5) | 11 (28.2) | 5 (20.0) | 6 (42.9) | .054 |
| Insomnia, mean (SD), *n* = 298 | 14.0 (7.2) | 13.6 (7.3) | 16.0 (6.4) | 16.2 (6.4) | 15.8 (6.6) | .865 |
| Traumatic life events, *n* = 312 |  |  |  |  |  |  |
| Number of, mean (SD) | 3.1 (2.6) | 2.9 (2.3) | 4.6 (3.3) | 4.9 (3.1) | 4.1 (3.6) | .438 |
| Physical abuse, *n* (%) | 126 (40.4) | 103 (38.0) | 23 (56.1) | 17 (63.0) | 6 (42.9) | .219 |
| Sexual abuse, *n* (%) | 90 (28.8) | 74 (27.3) | 16 (39.0) | 11 (40.7) | 5 (35.7) | .754 |
| Personality, mean (SD), *n* = 313 |  |  |  |  |  |  |
| Neuroticism | 53.2 (8.6) | 52.8 (8.4) | 55.9 (9.3) | 55.4 (8.7) | 56.7 (10.7) | .682 |
| Extraversion | 49.0 (7.1) | 48.6 (7.1) | 51.9 (6.4) | 51.5 (6.2) | 52.7 (6.9) | .562 |
| Aggressiveness | 50.6 (5.9) | 50.4 (6.0) | 51.4 (5.3) | 51.2 (5.8) | 51.8 (4.5) | .761 |
| Substance use disorder, *n* (%), *n* = 338 |  |  |  |  |  |  |
| 12 m prevalence | 12 (3.6) | 6 (2.0) | 6 (14.0) | 3 (10.7) | 3 (20.0) | .647 |
| Life time prevalence | 32 (9.5) | 23 (7.8) | 9 (20.9) | 6 (21.4) | 3 (20.0) | 1.00 |
| **COGNITIVE-BEHAVIOURAL VARIABLES** |  |  |  |  |  |  |
| Pain Catastrophising, mean (SD), *n* = 302 | 24.1 (12.7) | 23.2 (12.3) | 29.7 (13.7) | 27.7 (13.8) | 33.2 (13.3) | .218 |
| Fear of movement/(re) injury, mean (SD), *n* = 296 | 23.9 (7.6) | 23.4 (7.4) | 27.5 (7.9) | 28.0 (8.5) | 26.7 (6.9) | .625 |
| Pain self-efficacy, mean (SD), *n* = 306 | 6.0 (3.5) | 6.0 (3.5) | 5.9 (3.7) | 5.9 (3.9) | 5.9 (3.5) | .990 |
| Injustice, mean (SD), *n* = 302 | 22.8 (12.6) | 21.9 (12.4) | 29.0 (12.2) | 29.1 (12.1) | 28.7 (12.8) | .925 |
| **PHYSICAL CAPACITY** |  |  |  |  |  |  |
| Balance, mean (SD), *n* = 323 | 20.0 (6.4) | 19.8 (6.5) | 20.8 (5.5) | 20.0 (6.4) | 22.5 (3.2) | .347 |
| Walking speed (m/s), mean (SD), *n* = 311 | 1.1 (0.3) | 1.1 (0.3) | 1.1 (0.3) | 1.1 (0.4) | 1.2 (0.2) | .581 |

^a^ Differences in the n for each independent variable are due to incomplete responses or non-response.

^b^ Differences between individuals with mild P-OUD and individuals with moderate to severe P-OUD were examined with the student t-test or Mann-Whitney U test for continuous variables, and the chi-square test or the fisher’s exact test for dichotomous variables.

^c^ Self-rated health status was measured by EQ VAS 0-100.

^d^ Composite score, i.e., the mean of the worst pain, least pain, pain on average and pain right now.

^e^ The past 24 h.
